# IGFBP5 increases cell invasion and inhibits cell proliferation by EMT and Akt signaling pathway in Glioblastoma multiforme cells

**DOI:** 10.1186/s13008-020-00061-6

**Published:** 2020-02-27

**Authors:** Chengyuan Dong, Junwen Zhang, Sheng Fang, Fusheng Liu

**Affiliations:** 1 Brain Tumor Research Center, Beijing Neurosurgical Institute, Capital Medical University, Beijing, 100070 People’s Republic of China; 2 Department of Neurosurgery, Beijing Tiantan Hospital Affiliated to Capital Medical University, Beijing, 100070 People’s Republic of China; 3 Beijing Laboratory of Biomedical Materials, Beijing, 100070 People’s Republic of China

**Keywords:** GBM, IGFBP5, Akt, EMT, Invasion, Proliferation

## Abstract

**Background:**

Recurrence of Glioblastoma multiforme (GBM) seems to be the rule despite combination therapies. Cell invasion and cell proliferation are major reasons for recurrence of GBM. And insulin-like growth factor binding protein 5 (IGFBP5) is the most conserved of the IGFBPs and is frequently dysregulated in cancers and metastatic tissues.

**Results:**

By studying the human glioma tissues, we find that IGFBP5 expression associate to the histopathological classification and highly expressed in GBM. Using IGFBP5 mutants we demonstrate that knockdown of IGFBP5 inhibited cell invasion, whereas promoting cell proliferation in GBM cells. Mechanistically, we observed that promoting GBM cell proliferation by inhibiting IGFBP5 was associated with stimulating Akt (Protein kinase B) phosphorylation. However, IGFBP5 promote GBM cell invasion was related to the epithelial-to-mesenchymal transition (EMT). Furthermore, the Chinese Glioma Genome Altas (CGGA) database show that IGFBP5 is significantly increased in recurrent glioma and it predicted worse survival.

**Conclusions:**

The obtained results indicate that IGFBP5 has two sides in GBM—inhibiting cell proliferation but promoting cell invasion.

## Background

Glioblastoma multiforme (GBM) is the most common malignant and aggressive intracranial tumor in adults [1]. The average annual age-adjusted incidence rate of GBM was about 3–5/100,000/year [2, 3]. And the mean overall survival achieved with combination therapies only 14.6 months [4]. Currently, maximal safe neurosurgical resection accompanied by radiotherapy and chemotherapy are the standard treatment for GBM [5]. However, recurrence seems to be the rule despite combination therapies [6]. Considering that the entire tumor cannot be removed because GBM invades to surrounding tissues and underlie tumor repopulation, making inhibit growth and invasion of GBM as a critical target. But, the mechanisms of GBM cells invade the surrounding tissue are still unclear.

Epithelial-to-mesenchymal transition (EMT) has emerged as a regulator of invasive state in glioma [7]. EMT is process in which epithelial cells changes that culminates in a mesenchymal phenotype, characterized by cells with weak cell adhesions and enhanced the migratory capacity [8]. There are many factors have been described as potent to drive EMT [8, 9]. Recent studies have shown that IGFBP5 expression can influence the EMT process [10, 11]. However, the effect of IGFBP5 on EMT remains unclear in GBM.

Insulin-like growth factor 1 (IGF-1) is a member of the insulin superfamily of growth-promoting peptides and is the most abundant and ubiquitous polypeptide growth factor [12]. And IGF binding proteins (IGFBPs) comprise a family of six proteins that function as critical regulators of IGF1 bioavailability [13]. IGFBP5 is the most conserved of the IGFBPs and is frequently dysregulated in cancers and metastatic tissues [14]. Many studies have shown that IGFBP5 can suppress tumor proliferation and metastasis [15], while others have shown that IGFBP5 can function as an oncogene to promote tumor metastasis [16]. IGFBP5 has both IGF dependent and IGF independent regulatory effects on cell function. IGFBP5 can bind to conserved amino terminal domains of IGF1 and affect IGF1 receptor (IGF1R) signaling thereby regulating cell function, while the IGF-independent mechanism does not alter IGF1R signaling [17].

Despite increasing number of studies have shown that IGFBP5 has been associated with various types of cancers, its function in the progression of cancer is controversial. The expression level and functional differences of IGFBP5 in distinct cancer types and in the same tissue type suggests that there are still many mysteries about IGFBP5 [18]. In previous studies, few have provided mechanistic insights for IGFBP5 in GBM. Thus, it is imperative to determine the function of IGFBP5 in GBM.

In the present study, we found that IGFBP5 exerts different effects on the proliferation and invasion of GBM cells. Knockdown of IGFBP5 inhibited the migration and invasion of GBM cells, whereas promoting cell proliferation. Mechanistically, we uncovered an unexpected role of IGFBP5 in promoting cell invasion by regulating EMT, but inhibiting cell proliferation by suppressing the phosphorylation of AKT.

## Results

### IGFBP5 expression was upregulated in high grade glioma

**Fig. 1:**
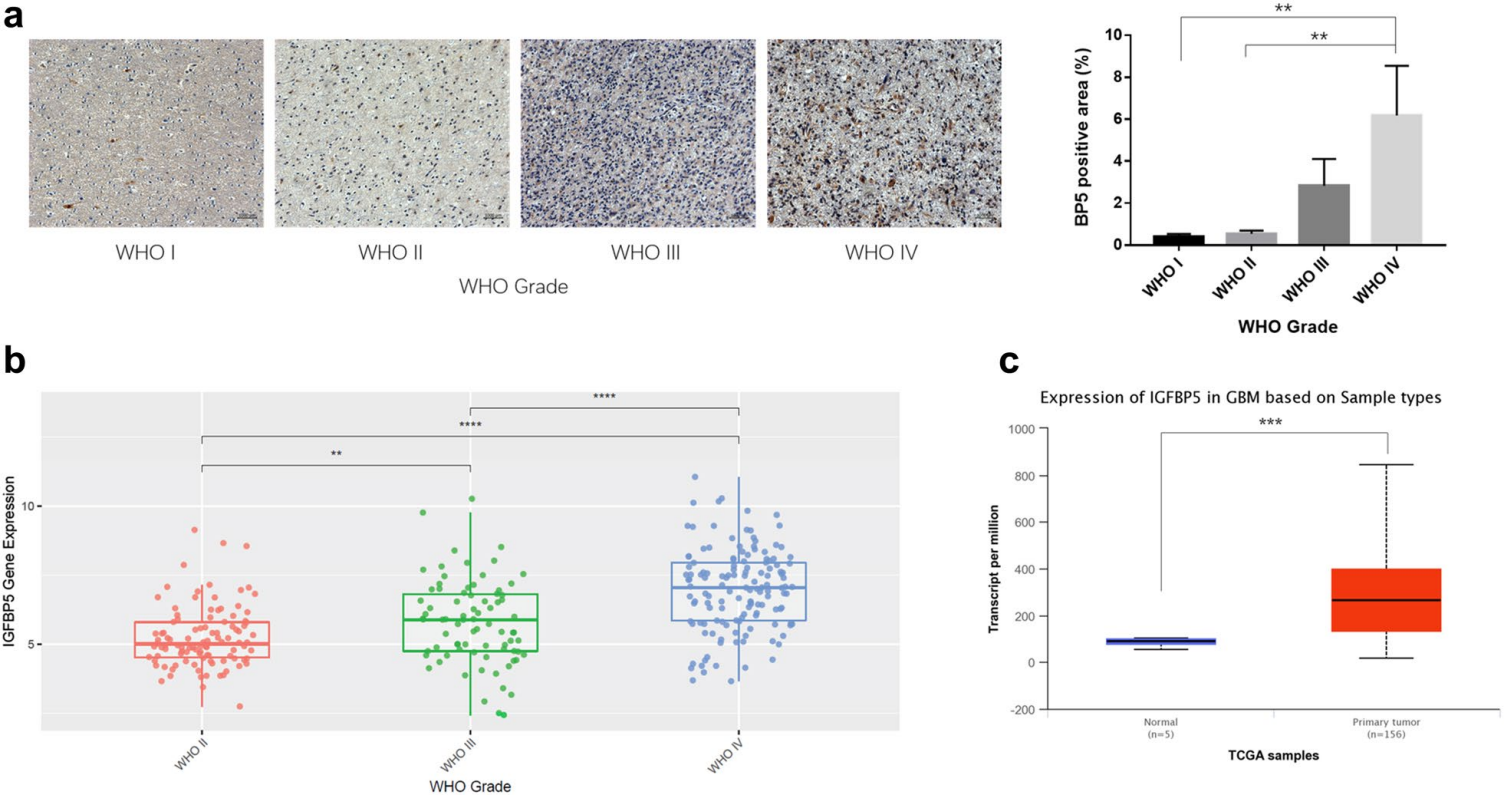
IGFBP5 expression was upregulated in high grade glioma. **a** Immunohistochemistry analysis showed that IGFBP5 expression clinically correlated with the progression of glioma. Scale bar = 1000 μm. **b** IGFBP5 expression is significantly upregulated along with the WHO grade in the CGGA database. **c** IGFBP5 expression is significantly upregulated in GBM contrasted to normal brain samples in the TCGA database. **P < 0.01, ***P < 0.001 and ****P < 0.0001

In order to analyze the expression of IGFBP5 in glioma, we stained of human glioma tissues with antibodies against IGFBP5, and the results indicated that IGFBP5 clinically correlated with the progression of glioma (Fig. 1a). We then analyzed The Cancer Genome Atlas (TCGA) database [19] and Chinese Glioma Genome Altas (CGGA) database (http://www.cgga.org.cn), similar results were obtained and found that GBM tumors with elevated expression of IGFBP5 (Fig. 1b, c). Taken together, these findings suggest that IGFBP5 is positively correlated with the progression of glioma.

### Short interfering RNA (siRNA) deplete IGFBP5

**Fig. 2:**
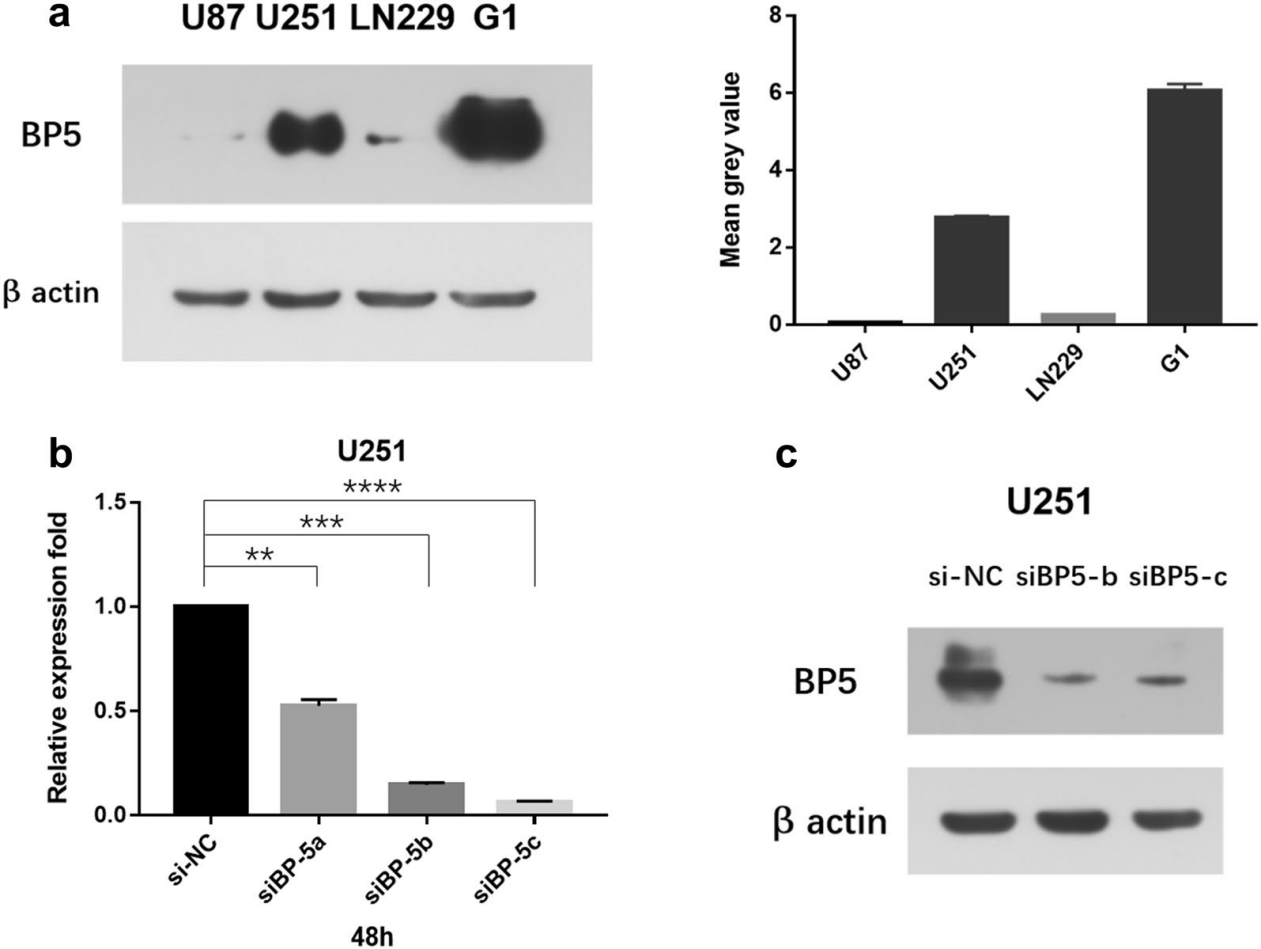
IGFBP5 expression levels in different cell lines and IGFBP5 were depleted by siRNA. **a** Western blot analysis demonstrated that U251 and G1 cell lines express high levels of IGFBP5 protein. **b** qPCR showed that siRNA of IGFBP5 downregulated the IGFBP5 expression in study group. **c** Western blot confirmed that IGFBP5 expression had been downregulated using siBP5-b and siBP5-c. **P < 0.01, ***P < 0.001 and ****P < 0.0001

To deepen study the functions of IGFBP5 in GBM, we transfected the human GBM cells with siRNA specifically targeting the IGFBP5. First, we used anti-IGFBP5 antibody to analyze the IGFBP5 expression levels in U87, U251, LN229 and one human GBM primary cell line (G1). As shown with Western blot (WB), the IGFBP5 expression level varied in different cell types, and U251 and G1 cells showed high IGFBP5 expression (Fig. 2a). Next, we transfected U251 cells with siRNA to deplete expression of IGFBP5. Compared to the control group, both mRNA and protein levels of IGFBP5 were downregulated in study group (Fig. 2b, c; Additional file 1: Fig. S1).

### Inhibit IGFBP5 represses GBM cell invasion

**Fig. 3:**
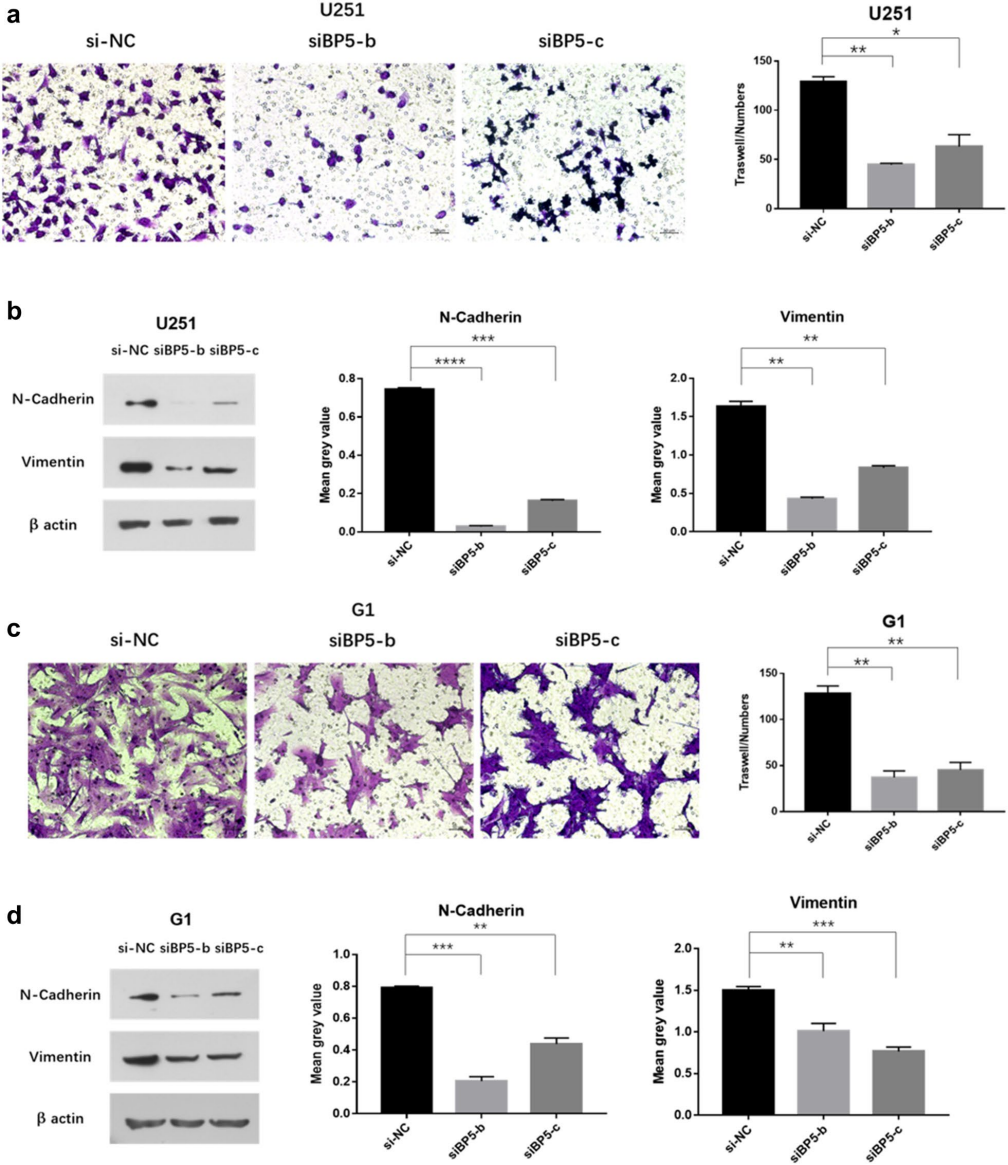
Inhibit IGFBP5 repressed GBM cell invasion. Matrigel cell invasion assay showed that knockdown of IGFBP5 inhibited the invasion ability of U251 (**a**) and G1 (**c**) cells. Scale bar = 50 μm. **b**, **d** Western blot confirmed the reduced proteins’ levels of N-cadherin and Vimentin in siBP5 cells (U251 and G1) relative to control. *P < 0.05, **P < 0.01, ***P < 0.001 and ****P < 0.0001

To investigate the role of IGFBP5 in GBM progression, we transfected the GBM cell lines U251 and G1 with IGFBP5 siRNAs, which enabling downregulated expression of IGFBP5. Then, we assessed the effects of IGFBP5 on cell invasion using Matrigel cell invasion assay. Our results showed that downregulated expression of IGFBP5 can significantly repressed GBM cell invasion (Fig. 3a, c).

Because previously study showed that N-cadherin enhanced glioma migration and metastasis [20]. And Vimentin is a marker of cellular EMT, a phenomenon associated with GBM invasion and metastatic [21]. We speculated that N-cadherin and Vimentin proteins are downregulated by depleting IGFBP5 in GBM. To examine this possibility, we assayed the N-cadherin and Vimentin proteins levels using WB after transfected siRNA of IGFBP5 into GBM cells. The results were found that downregulated expression of IGFBP5 can decrease the expression of N-cadherin and Vimentin proteins (Fig. 3b, d). These data strongly suggest inhibit IGFBP5 represses GBM cell invasion.

### Inhibit IGFBP5 promotes GBM cell proliferation

**Fig. 4:**
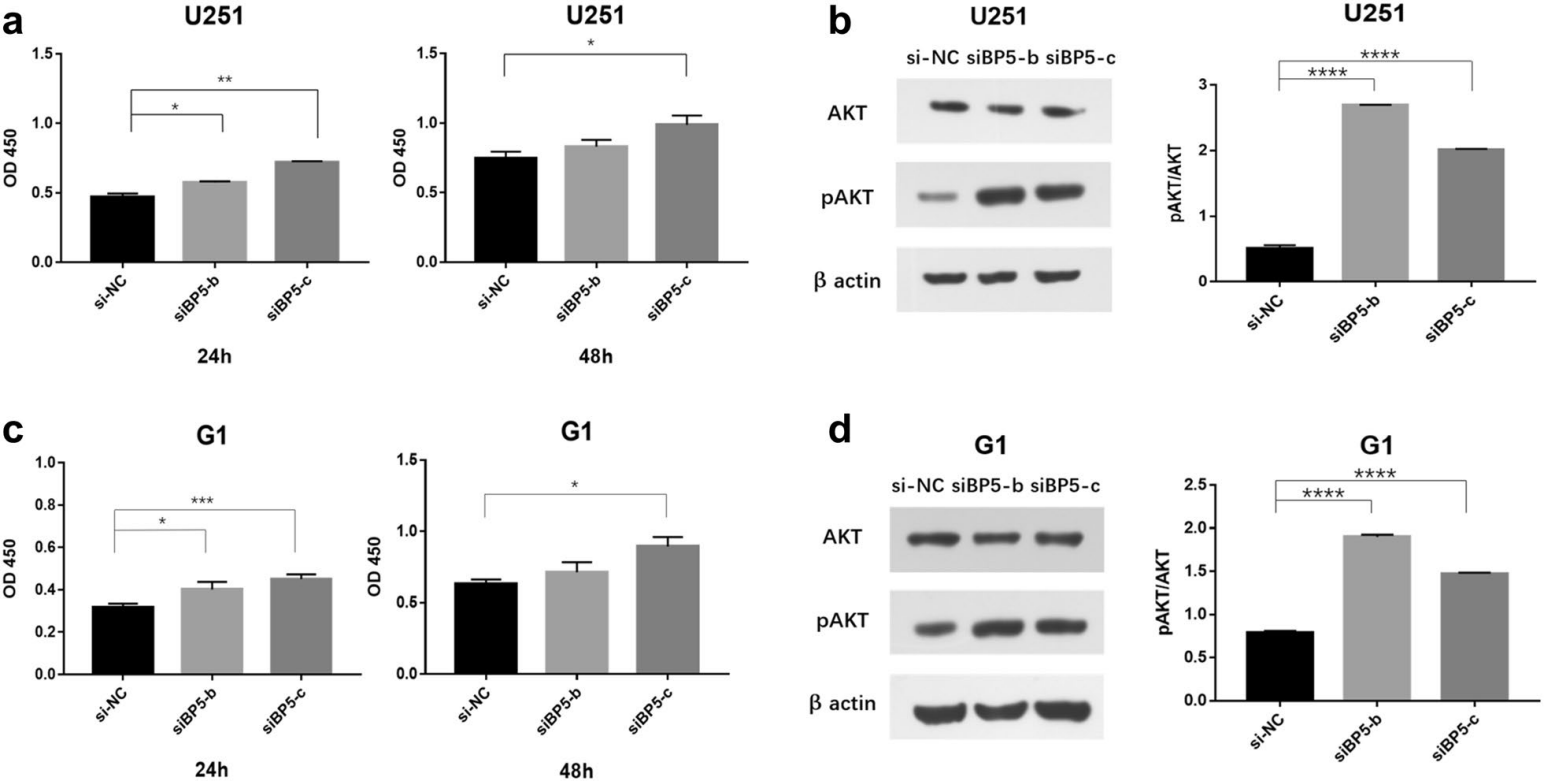
Inhibit IGFBP5 promotes GBM cell proliferation. **a**, **c** CCK-8 assay were used to explore the U251 and G1 cell proliferation after transfected with siBP5 or si-NC for 24 h or 48 h. **b**, **d** Akt and pAkt were detected by Western blot analysis and β actin was used as an internal control, the corresponding semi-quantitative analysis of pAkt(Ser473)/Akt ration was based on optical density with ImageJ software. *P < 0.05, **P < 0.01, ***P < 0.001 and ****P < 0.0001

Next, we wished to determine whether knockdown IGFBP5 can also repressed GBM cell proliferation. To this end, we used Cell Counting Kit-8 (CCK-8) assay to assess the effect of IGFBP5 on cell proliferation. Surprisingly, the experiment turned out to be completely contrary to our suspected. Down-regulation of IGFBP5 actually promoted cell proliferation (Fig. 4a, c).

Because IGFBP5 had been shown to stimulate proliferation is related to the PI3K (phosphoinositol-3-kinase)/Akt pathway in cancers cells [22], we examined the Akt and pAkt proteins levels using WB after IGFBP5 silencing. We observed IGFBP5 silencing result in an increase in pAkt protein levels both in U251 and G1 cell lines (Fig. 4b, d; Additional file 2: Fig.  S2). Together, our results indicate that inhibit IGFBP5 promotes GBM cell proliferation.

### IGFBP5 expression levels are significantly elevated in recurrent glioma compared with the primary glioma

**Fig. 5:**
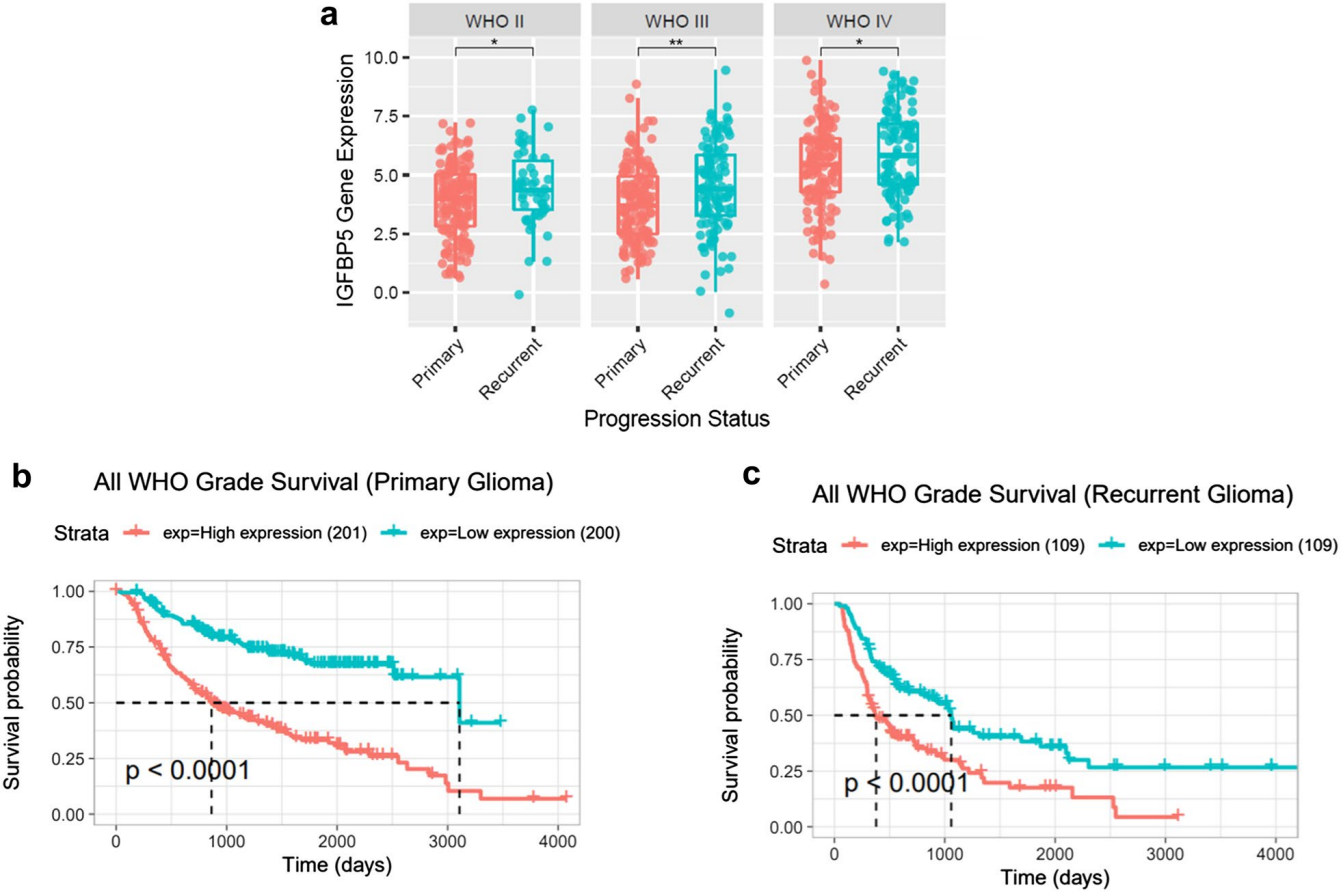
Recurrent glioma expressed a higher level of IGFBP5, and high expression of IGFBP5 result in poor prognosis. **a** IGFBP5 was significantly increased in recurrent glioma in the CGGA database. **b**, **c** IGFBP5 predicted worse survival both in primary glioma and recurrent glioma in the CGGA database. *P < 0.05, **P < 0.01

Since IGFBP5 can promote GBM cell invasion, we speculated that recurrent glioma IGFBP5 expression levels should be higher than primary glioma. To test this, we evaluated the expression of IGFBP5 in CGGA database. Consistent with our speculate, recurrent glioma expressed a higher level of IGFBP5 than primary glioma (Fig. 5a). And the database show that high expression of IGFBP5 in glioma or recurrent glioma resulted in poor prognosis (Fig. 5b, c).

## Discussion

The IGFBPs family includes many subtypes. Many previous studies indicate that IGFBPs can promote tumor growth but can also function as anti-oncogenes [17]. IGFBP2 has been demonstrated to show promoted tumorigenicity in various cancer types [1, 23, 24]. And in high quality glioma studies, IGFBP-2 showed non conflicting results in predictive biomarkers [25]. However, other IGFBPs have differ roles among different tumor circumstances [17]. IGFBP5 as the most conserved of the IGFBPs, its different domains have different effects on tumorigenicity and metastasis of different human cancers [26, 27]. IGFBP5 has been involved in regulating the growth and development of various types of cancers [18], but few studies have provided any deep understanding of IGFBP5 on GBM. We have identified a different role of IGFBP5 in the induction of GBM cell proliferation and invasion that could provide a new insight into the treatment of GBM.

In addition to reports that the expression of IGFBP5 clinically correlates with the histologic grade of GBM, our study further identified IGFBP5 can promote GBM cell invasion and elucidated that its promoting effect was related to the EMT. EMT is essential for enabling tumor cells to invade surrounding tissues and eventually disseminate to distant sites [28, 29]. During EMT, E-cadherin switch to the N-cadherin [30], and the expression of mesenchymal proteins, N-cadherin and vimentin are increased [21, 31]. These previously published studies are consistent with the results presented here. These results also explain why the expression of IGFBP5 in recurrent GBM is higher than that in primary GBM (Fig. 5a). And GBM recurrence is nearly universal and no current regimen can overcome [32], whereas the biology of recurrent glioblastoma remains largely unknown. Our study suggests that EMT could be a therapeutic target for recurrent GBM.

A characteristic feature of GBM is rapid growth [33]. In this study, we investigated the effect of IGFBP5 on GBM proliferation. Previous studies on the role of IGFBP5 in the proliferation of cancer cells are inconsistent. IGFBP-5 expression is an important mediator of pancreatic cancer cell growth [34]. And IGFBP5 can reversed the anti-proliferation effects of miR-204-5p in papillary thyroid carcinoma [35]. On the other hand, IGFBP5 can suppress the gastric cancer cell growth [15]. And overexpression of IGFBP5 inhibits cell proliferation in human osteosarcoma cell lines [36] and human melanoma cell line [10]. However, our study found that inhibiting IGFBP5 can promote GBM cell proliferation by stimulating Akt phosphorylation. Activation of Akt is an important cell proliferation signal when treated with IGFBP5 [22], but we showed that down-regulation of IGFBP5 induced an activation of phosphorylation of Akt in GBM.

Thus, we show for the first time that IGFBP5 has two sides in GBM—inhibiting cell proliferation but promoting cell invasion. This result leads us to speculate that GBM cells tend to proliferation rather than invasion when IGFBP5 is absent. As a recent study shows, E-cadherin acts as a survival factor in invasive ductal carcinomas during the systemic dissemination and seeding phases of metastasis [37]. Our results support the concept of cancer cell invasion and proliferation is a complex and variable process. And there are many mysteries that remain to be solved about the IGFBP5 protein.

## Conclusions

In summary, our study demonstrated that IGFBP5 has a dual role in GBM—inhibiting cell proliferation but promoting cell invasion, which involve Akt and EMT signaling pathway. This result leads us to believe that the treatment of GBM requires targeting tumor proliferation and invasion, respectively. And further studies are needed to dissect the structural and molecular basis of IGFBP5 action in GBM.

## Methods

### Cell culture and tissue samples

The human GBM cell lines U87, U251, LN229 were purchased from Cell Bank of Peking Union Medical College (Beijing, China). And the human primary GBM cell line G1 was established from the GBM tissue of an untreated 37-year-old male patient. Cells were cultured in DMEM (Invitrogen, Carlsbad, CA, USA) with 10% fetal bovine serum (FBS, EVERY GREEN, Zhejiang, China), 2 mM l-glutamine, 100 U/ml penicillin and 100 μg/ml streptomycin at 37 °C in an environment of 5% CO_2_.

The acquisition of tissue samples was approved by the institutional review board of Beijing Tiantan Hospital affiliated to Capital Medical University. And informed consent was given by all patients examined. At least three experienced pathologists confirmed the histological diagnoses.

### Immunohistochemistry (IHC)

Immunohistochemistry (IHC) experiments were performed as previously described [38]. Anti-IGFBP5 (1:500, sc-515184, Santa Cruz, CA, USA) was used to measure the protein expression levels of different grade gliomas. For image acquisitions, three random fields were obtained in each slide by Zeiss Axio-Imager.M2 (Carl Zeiss Microscopy GmbH, Oberkochen, Germany). All tissue slides were reviewed by a blinded pathologist confirmed the histological diagnoses. And the area percentage of staining in a 200× magnification were analyzed by ImageJ software (Version 1.52, Bethesda, MD, USA).

### RNA interference, RNA extraction, and quantitative real-time PCR

Quantitative real-time PCR (qPCR) was used to assess the mRNA levels of IGFBP5 in si-negative control (si-NC) and si-IGFBP5 (siBP5) cells according to the procedures as described before [39]. The primers used for qPCR were as follows: IGFBP5, forward 5′-CGGGGTTTGCCTCAACGAA -3′, reverse 5′TCTTGGGGGAGTAGGTCTCCT-3′; GAPDH, forward 5′-CTGCACCACCAACTGCTTAGC-3′, reverse 5′-CTTCACCACCTTCTTGATGTC 3′.

Three siRNAs specifically targeting IGFBP5 were designed and provided by Guangzhou RIBOBIO Co., Ltd (Guangzhou, China). siRNA sequences were as follows: siBP5-a: GCCCAATTGTGACCGCAAA; siBP5-b: GCCTCAACGAAAAGAGCTA; siBP5-c: GACCGCAAAGGATTCTACA. U251 and G1 cells were seeded in 6-well plates and then transfected with 75 pmol siRNAs by using Lipofectamine 3000 (Invitrogen, Carlsbad, CA, USA) according to the manufacturer’s instructions. The cells were collected 24–72 h post-transfection for qPCR or WB analysis.

### Western blots analysis

Western blots analysis was used to assess protein expression levels in si-NC and siBP5 cells, and performed as previously described [39]. The following primary antibodies were used: anti-IGFBP5 (1:500, sc-515148, Santa Cruz), anti-β actin (1:5000, A5441, Sigma-Aldrich, Oslo, Norway), anti-Vimentin (1:1000, 5741, Cell Signaling Technology (CST), Danvers, MA, USA), anti-N-cadherin (1:1000, 13116, CST), anti-AKT(pan) (1:1000, 4691, CST) anti-pAKT (Thr308) (1:1000, 13038, CST) and anti-pAKT (Ser473) (1:1000, 4060, CST).

### Transwell invasion assays

U251 and G1 cells transfected with si-NC or siBP5-b/c were seeded at 5 × 10^4^ per well onto the upper Transwell chambers (8-μm pore size, Corning), which were pre-treated with the growth factor reduced basement membrane matrix (Geltrex, A1413202, Invitrogen) evenly and incubated at 37 °C for 30 min. And 10% FBS-containing medium was placed into the lower chamber. After incubation under 37 °C in 5% CO_2_ for 12 h, the invasion cells through the pores were fixed with 4% paraformaldehyde and stained with crystal violet. And the non-invasion cells at the upper chambers were removed. Then penetrated cells were quantified by ImageJ.

### Cell proliferation assay

To explore the effect of IGFBP5 on GBM cell proliferation, a CCK8 (Dojindo, Kumamoto, Japan) assay was performed following the manufacturer’s guidelines as described previously [39].

### Statistical analysis

Statistical significance was assessed by one-way ANOVA with Bonferroni correction for multiple comparisons using Prism 7 (GraphPad Software Inc. CA, USA). All experiments were performed at least three times. Data were expressed as mean ± standard deviation (SD). Differences between samples were considered significant at *P < 0.05, **P < 0.01, ***P < 0.001 and ****P < 0.0001 (Additional file 1: Fig. S1, Additional file 2: Fig. S2).

## Supplementary information

**Additional file 1: Fig. S1.** IGFBP5 were depleted by siRNA. qPCR showed that siRNA of IGFBP5 downregulated the IGFBP5 expression in study group. **P < 0.01, ***P < 0.001 and ****P < 0.0001.

**Additional file 2: Fig. S2.** IGFBP5 silencing increase pAkt protein expression. Akt and pAkt were detected by Western blot analysis and β actin was used as an internal control, the corresponding semi-quantitative analysis of pAkt(Thr308)/Akt ration was based on optical density with ImageJ software. **P < 0.01, ***P < 0.001.

## Data Availability

Not applicable.

## Abbreviations

GBM: Glioblastoma multiforme
IGFBP5: Insulin-like growth factor binding protein 5
EMT: Epithelial-to-mesenchymal transition
CGGA: Chinese Glioma Genome Altas
IGF-1: Insulin-like growth factor 1
IGF1R: IGF1 receptor
TCGA: The Cancer Genome Atlas
siRNA: Short interfering RNA
WB: Western blot
PI3K: Phosphoinositol-3-kinase
Akt: Protein kinase B
CCK-8: Cell Counting Kit-8
qPCR: Quantitative real-time PCR

## References

[1] Liu Y, Li F, Yang YT, Xu XD, Chen JS, Chen TL (2019). IGFBP2 promotes vasculogenic mimicry formation via regulating CD144 and MMP2 expression in glioma. Oncogene.

[2] Brodbelt A, Greenberg D, Winters T, Williams M, Vernon S, Collins VP, National Cancer Information Network Brain Tumour G (2015). Glioblastoma in England: 2007–2011. Eur J Cancer..

[3] Ostrom QT, Cioffi G, Gittleman H, Patil N, Waite K, Kruchko C (2019). CBTRUS statistical report: primary brain and other central nervous system tumors diagnosed in the United States in 2012–2016. Neuro Oncol.

[4] Das S, Marsden PA (2013). Angiogenesis in glioblastoma. N Engl J Med.

[5] Cai J, Chen Q, Cui Y, Dong J, Chen M, Wu P (2018). Immune heterogeneity and clinicopathologic characterization of IGFBP2 in 2447 glioma samples. Oncoimmunology.

[6] Alifieris C, Trafalis DT (2015). Glioblastoma multiforme: pathogenesis and treatment. Pharmacol Ther.

[7] Iser IC, Pereira MB, Lenz G, Wink MR (2017). The epithelial-to-mesenchymal transition-like process in glioblastoma: an updated systematic review and in silico investigation. Med Res Rev.

[8] De Craene B, Berx G (2013). Regulatory networks defining EMT during cancer initiation and progression. Nat Rev Cancer.

[9] Muhammad N, Bhattacharya S, Steele R, Phillips N, Ray RB (2017). Involvement of c-Fos in the promotion of cancer stem-like cell properties in head and neck squamous cell carcinoma. Clin Cancer Res.

[10] Wang J, Ding N, Li Y, Cheng H, Wang D, Yang Q (2015). Insulin-like growth factor binding protein 5 (IGFBP5) functions as a tumor suppressor in human melanoma cells. Oncotarget.

[11] Vijayan A, Guha D, Ameer F, Kaziri I, Mooney CC, Bennett L (2013). IGFBP-5 enhances epithelial cell adhesion and protects epithelial cells from TGFbeta1-induced mesenchymal invasion. Int J Biochem Cell Biol.

[12] Blumenthal S (2010). From insulin and insulin-like activity to the insulin superfamily of growth-promoting peptides: a 20th-century odyssey. Perspect Biol Med.

[13] Wang H, Wang H, Zhang W, Fuller GN (2006). Overexpression of IGFBP5, but not IGFBP3, correlates with the histologic grade of human diffuse glioma: a tissue microarray and immunohistochemical study. Technol Cancer Res Treat.

[14] Tripathi G, Salih DA, Drozd AC, Cosgrove RA, Cobb LJ, Pell JM (2009). IGF-independent effects of insulin-like growth factor binding protein-5 (Igfbp5) in vivo. FASEB J.

[15] Zhang L, Li W, Cao L, Xu J, Qian Y, Chen H (2019). PKNOX2 suppresses gastric cancer through the transcriptional activation of IGFBP5 and p53. Oncogene.

[16] Zhang T, Guo J, Gu J, Wang Z, Wang G, Li H (2019). Identifying the key genes and microRNAs in colorectal cancer liver metastasis by bioinformatics analysis and in vitro experiments. Oncol Rep.

[17] Baxter RC (2014). IGF binding proteins in cancer: mechanistic and clinical insights. Nat Rev Cancer.

[18] Gullu G, Karabulut S, Akkiprik M (2012). Functional roles and clinical values of insulin-like growth factor-binding protein-5 in different types of cancers. Chin J Cancer.

[19] Chandrashekar DS, Bashel B, Balasubramanya SAH, Creighton CJ, Ponce-Rodriguez I, Chakravarthi B (2017). UALCAN: a portal for facilitating tumor subgroup gene expression and survival analyses. Neoplasia.

[20] Noh MG, Oh SJ, Ahn EJ, Kim YJ, Jung TY, Jung S (2017). Prognostic significance of E-cadherin and N-cadherin expression in Gliomas. BMC Cancer.

[21] Nowicki MO, Hayes JL, Chiocca EA, Lawler SE (2019). Proteomic analysis implicates vimentin in glioblastoma cell migration. Cancers.

[22] Sureshbabu A, Okajima H, Yamanaka D, Tonner E, Shastri S, Maycock J (2012). IGFBP5 induces cell adhesion, increases cell survival and inhibits cell migration in MCF-7 human breast cancer cells. J Cell Sci.

[23] Uzoh CC, Holly JM, Biernacka KM, Persad RA, Bahl A, Gillatt D (2011). Insulin-like growth factor-binding protein-2 promotes prostate cancer cell growth via IGF-dependent or -independent mechanisms and reduces the efficacy of docetaxel. Br J Cancer.

[24] Gao S, Sun Y, Zhang X, Hu L, Liu Y, Chua CY (2016). IGFBP2 activates the NF-kappaB pathway to drive epithelial–mesenchymal transition and invasive character in pancreatic ductal adenocarcinoma. Cancer Res.

[25] Pierscianek D, Ahmadipour Y, Oppong MD, Rauschenbach L, Kebir S, Glas M (2019). Blood-based biomarkers in high grade gliomas: a systematic review. Mol Neurobiol.

[26] Luther GA, Lamplot J, Chen X, Rames R, Wagner ER, Liu X (2013). IGFBP5 domains exert distinct inhibitory effects on the tumorigenicity and metastasis of human osteosarcoma. Cancer Lett.

[27] Akkiprik M, Hu L, Sahin A, Hao X, Zhang W (2009). The subcellular localization of IGFBP5 affects its cell growth and migration functions in breast cancer. BMC Cancer.

[28] Tsuji T, Ibaragi S, Hu GF (2009). Epithelial–mesenchymal transition and cell cooperativity in metastasis. Cancer Res.

[29] Thiery JP (2002). Epithelial–mesenchymal transitions in tumour progression. Nat Rev Cancer.

[30] Casal JI, Bartolome RA (2019). Beyond N-cadherin, relevance of cadherins 5, 6 and 17 in cancer progression and metastasis. Int J Mol Sci.

[31] Appolloni I, Barilari M, Caviglia S, Gambini E, Reisoli E, Malatesta P (2015). A cadherin switch underlies malignancy in high-grade gliomas. Oncogene.

[32] Osuka S, Van Meir EG (2017). Overcoming therapeutic resistance in glioblastoma: the way forward. J Clin Invest.

[33] Ahn SH, Park H, Ahn YH, Kim S, Cho MS, Kang JL (2016). Necrotic cells influence migration and invasion of glioblastoma via NF-kappaB/AP-1-mediated IL-8 regulation. Sci Rep.

[34] Johnson SK, Haun RS (2009). Insulin-like growth factor binding protein-5 influences pancreatic cancer cell growth. World J Gastroenterol.

[35] Liu L, Wang J, Li X, Ma J, Shi C, Zhu H (2015). MiR-204-5p suppresses cell proliferation by inhibiting IGFBP5 in papillary thyroid carcinoma. Biochem Biophys Res Commun.

[36] Su Y, Wagner ER, Luo Q, Huang J, Chen L, He BC (2011). Insulin-like growth factor binding protein 5 suppresses tumor growth and metastasis of human osteosarcoma. Oncogene.

[37] Padmanaban V, Krol I, Suhail Y, Szczerba BM, Aceto N, Bader JS (2019). E-cadherin is required for metastasis in multiple models of breast cancer. Nature.

[38] Zhou Y, Jin G, Mi R, Dong C, Zhang J, Liu F (2014). The methylation status of the platelet-derived growth factor-B gene promoter and its regulation of cellular proliferation following folate treatment in human glioma cells. Brain Res.

[39] Dong C, Mi R, Jin G, Zhou Y, Zhang J, Liu F (2015). Identification of the proliferative effect of Smad2 and 3 in the TGF beta2/Smad signaling pathway using RNA interference in a glioma cell line. Mol Med Rep.

