# Supplementary figures and images for: IGFBP5 increases cell invasion and inhibits cell proliferation by EMT and Akt signaling pathway in Glioblastoma multiforme cells
Source: Cell Div. 2020 Feb 27;15:4. doi: 10.1186/s13008-020-00061-6 (PMC7047354; doi:10.1186/s13008-020-00061-6)

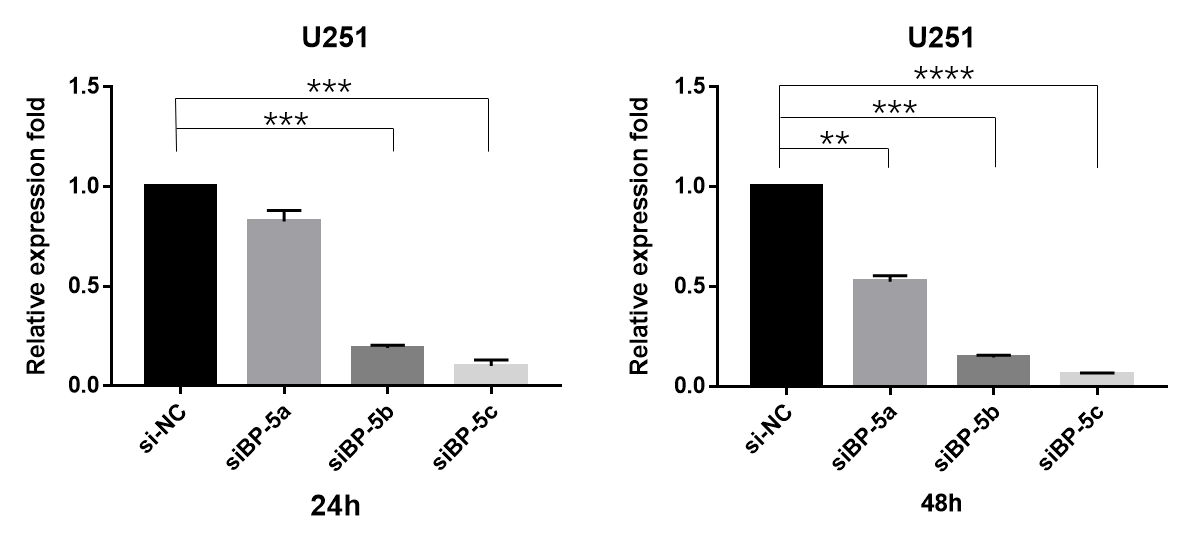

Supplement: Supplementary file 1 — Additional file 1: Fig. S1. IGFBP5 were depleted by siRNA. qPCR showed that siRNA of IGFBP5 downregulated the IGFBP5 expression in study group. **P < 0.01, ***P < 0.001 and ****P < 0.0001. [file 13008_2020_61_MOESM1_ESM.tif]

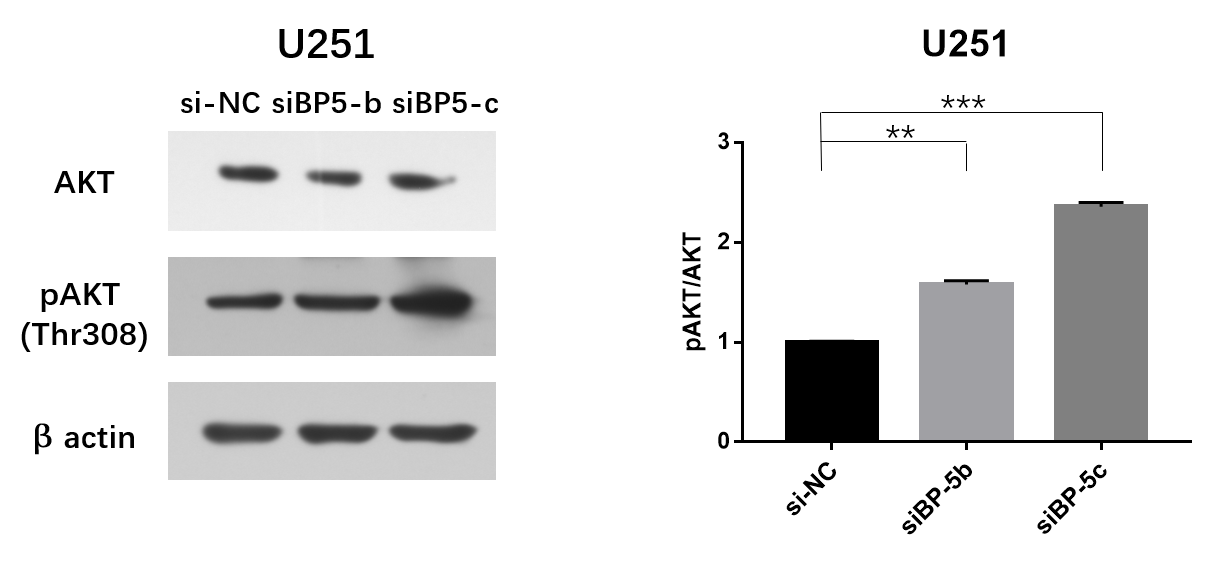

Supplement: Supplementary file 2 — Additional file 2: Fig. S2. IGFBP5 silencing increase pAkt protein expression. Akt and pAkt were detected by Western blot analysis and β actin was used as an internal control, the corresponding semi-quantitative analysis of pAkt(Thr308)/Akt ration was based on optical density with ImageJ software. **P < 0.01, ***P < 0.001. [file 13008_2020_61_MOESM2_ESM.tif]
